# Changepoint Detection in Noisy Data Using a Novel Residuals Permutation-Based Method (RESPERM): Benchmarking and Application to Single Trial ERPs

**DOI:** 10.3390/brainsci12050525

**Published:** 2022-04-21

**Authors:** Werner Sommer, Katarzyna Stapor, Grzegorz Kończak, Krzysztof Kotowski, Piotr Fabian, Jeremi Ochab, Anna Bereś, Grażyna Ślusarczyk

**Affiliations:** 1 Department of Psychology, Humboldt-University of Berlin, 10099 Berlin, Germany; werner.sommer@cms.hu-berlin.de; 2 Department of Psychology, Zhejiang Normal University, Jinhua 321000, China; 3 Department of Applied Informatics, Silesian University of Technology, 44-100 Gliwice, Poland; kotowski.polsl@gmail.com (K.K.); piotr.fabian@polsl.pl (P.F.); 4 Department of Statistics, Econometrics and Mathematics, University of Economics in Katowice, 40-287 Katowice, Poland; grzegorz.konczak@ue.katowice.pl; 5 Department of Theory of Complex Systems, Jagiellonian University, 30-348 Krakow, Poland; jeremi.ochab@uj.edu.pl; 6 Department of Cognitive Neuroscience and Neuroergonomics, Jagiellonian University, 30-348 Krakow, Poland; a.beres@uj.edu.pl; 7 Department of Design and Computer Graphics, Jagiellonian University, 30-348 Krakow, Poland; gslusarc@uj.edu.pl

**Keywords:** noisy time series, event-related potentials, changepoint detection, segmented method, permutation method

## Abstract

An important problem in many fields dealing with noisy time series, such as psychophysiological single trial data during learning or monitoring treatment effects over time, is detecting a change in the model underlying a time series. Here, we present a new method for detecting a single changepoint in a linear time series regression model, termed residuals permutation-based method (RESPERM). The optimal changepoint in RESPERM maximizes Cohen’s effect size with the parameters estimated by the permutation of residuals in a linear model. RESPERM was compared with the SEGMENTED method, a well-established and recommended method for detecting changepoints, using extensive simulated data sets, varying the amount and distribution characteristics of noise and the location of the change point. In time series with medium to large amounts of noise, the variance of the detected changepoint was consistently smaller for RESPERM than SEGMENTED. Finally, both methods were applied to a sample dataset of single trial amplitudes of the N250 ERP component during face learning. In conclusion, RESPERM appears to be well suited for changepoint detection especially in noisy data, making it the method of choice in neuroscience, medicine and many other fields.

## 1. Introduction

Changepoint analysis is important in many fields, including medical research and cognitive neuroscience, but also finance, economics, quality control, and genomics. In general, changepoint detection refers to the problem of finding changes in the underlying model of a signal or time series [1]. Hence, the changepoint detection problem can be formulated in different models and there are numerous approaches to examine these models, for example maximum-likelihood estimation, Bayesian estimation, piecewise regression, quasi-likelihood and non-parametric regression, and grid-searching. Changepoint detection can be applied in real-time or offline, after all samples have been received. The changepoint regression problem was first described by Chow [2]; a detailed review of subsequent studies on estimating the location of changepoints, as well as the classification and evaluation of different methods, can be found in [1,3].

Here, we aimed to locate changepoints in a linear regression model. *Changepoint regressions*, where the relationship between the response and the explanatory variables (i.e., the regressors) is piecewise linear have been termed, for example, “segmented” [4], “broken-line” [5], or “structural change” regression. Piecewise linear changepoint regression models come in continuous or discontinuous form, where regression lines with different slopes are connected at the changepoints or may jump to a different level at the changepoint(s), respectively.

The main contribution of the present paper is a new single-changepoint detection method based on a specific optimization criterion, Cohen’s effect size, estimated by the permutation of residuals, termed residuals permutation-based method (RESPERM). Using simulated data, we demonstrate lower mean square error (MSE) and less bias using RESPERM than the well-established and frequently recommended SEGMENTED method [6], especially for noisy data such as EEG signals. We apply both methods to time series of single trial ERP amplitudes during face learning (e.g., [7,8]).

In the following sections, we present the general model for changepoint regression and shortly describe SEGMENTED. In Section 1.3, we explain the relevance of changepoint detection to singe trial ERP information from EEG data. Section 2.1 and Section 2.2 describe the proposed RESPERM method and the Monte Carlo simulation setup for its verification and comparison with SEGMENTED, respectively. In Section 2.3, we shortly describe a sample EEG experiment for face learning [8]. Section 3 presents the results of the simulation study and the application of both methods to the sample EEG data, followed by a general discussion and conclusions in Section 4.

### 1.1. The Changepoint Regression Model

We consider models where changepoints are defined in terms of one regressor, denoted *x*. Model (1) considers a regressor *x* involving a changepoint; however, other regressors can also be included in the model. The general model of changepoint regression with J changepoints (J + 1 regimes) can be written as:

(1) $$y_{i}=\{\begin{array}{c} \;\;\;\beta_{01}+\beta_{11}x_{i}+\varepsilon_{i1}\;,\;x_{i}<chp_{1}\\ \;\;\;\beta_{02}+\beta_{12}x_{i}+\varepsilon_{i2}\;,\;chp_{1}<x_{i}\leq chp_{2}\\ \;\;\;\;\;\;\vdots \\ \;\;\;\beta_{0j}+\beta_{1j}x_{i}+\varepsilon_{ij}\;,\;chp_{j-1}<x_{i}\leq chp_{j}\\ \;\;\;\;\;\;\vdots \\ \beta_{0\left(J+1\right)}+\beta_{1\left(J+1\right)}x_{i}+\varepsilon_{i\left(J+1\right)}\;,\;chp_{J}<x_{i} \end{array}\;$$

where i=1,…, n are observations, n is the total sample size, $chp_{j},\;\;j=1,\ldots ,\;J$ are the changepoint parameters for the regressor *x*, which satisfy $chp_{1}<chp_{2}<\cdots <chp_{J}$.

$\varepsilon_{ij}$ are independent, identically distributed random variables (e.g., error or noise), with means of zero and variances $\sigma_{j}^{2},\;j=1,\ldots ,\;J$. The changepoint locations $chp_{j}$ are the unknown parameters to be estimated, whereas the number of changepoints in the observed sample is assumed to be known. Model (1) also assumes that regressor *x* can be ordered (i.e., partitioned by the changepoint $chp_{j}$) and a sufficient number of observations is contained in each regime for reliable estimation and inference. It is up to the user to determine what is “sufficient”, but a rule of thumb may be to ensure at least 10 observations in each regime. 

The model above applies to both continuous piecewise and discontinuous changepoints. To enforce connected regression lines, the regression parameters in model (1) must be constrained:

(2) $$\beta_{0j}+\beta_{1j}chp_{j}=\beta_{0\left(j+1\right)}+\beta_{1\left(j+1\right)}chp_{j+1}$$

Many methods aim to detect the location of unknown changepoints and estimate the regression model (1). A frequently recommended and approved method for changepoint regression is the *segmented regression* method by Muggeo [6,9] (termed here SEGMENTED), which will use as a benchmark for the proposed new RESPERM method.

### 1.2. SEGMENTED Method

SEGMENTED [9] allows to detect multiple unknown changepoints but is restricted to continuous regression lines. We briefly describe this method for the single changepoint model (J = 1) with location in chp. The model (1) with constraints (2) for the segmented regression can be estimated iteratively via the following linear function of predictors:

(3) $$\beta_{0}+\beta_{11}x_{i1}+\left(\beta_{12}-\beta_{11}\right)\left(x_{i1}-chp_{0}\right)I\left(x_{i1}>chp_{0}\right)-\gamma I\left(x_{i1}>chp_{0}\right)$$

where I(A) is an indicator function for an event A, $chp_{0}$ is an initial estimate for the changepoint and γ is a re-parametrization of $chp_{0}$ that appears as a linear and continuously valued parameter, facilitating the estimation procedure. Muggeo [6] recommended maximum likelihood (ML) under Gaussian errors with constant variance across regimes (homoscedasticity). The model enables for simultaneous ML inference on all model parameters, including the changepoint location. According to Google Scholar, SEGMENTED was cited in over 1500 articles in a wide variety of scientific domains, from developmental psychology [10] to epidemiology [11].

### 1.3. Application to ERPs

EEG reflects the mass-action of mostly post-synaptic potentials in different brain states and processes. Specific activities related to defined events can be derive from event-related potentials (ERP) by time locking the EEG to stimuli or responses. ERPs contain a number of components (most simply peaks or troughs) that are sensitive to certain mental operations, for example, the perceptual and mnemonic processing of faces [12] or decision making [13]. Due to the low signal to noise ratio of ERPs relative to the background EEG [14], usually, the event-locked signal is averaged across many repetitions. Averaging efficiently reduces the randomly fluctuating background EEG towards increasing smaller values relative to the signal ERP [15]. However, averaging can be misleading because it might not reflect the actual brain dynamics (e.g., [16]) and when dynamic changes over a few trials are of interest, it may be close to useless. There are other methods for decreasing the noise in single trial EEG signal like low-pass filters, wavelet analysis, artifacts reduction, or re-referencing. However, these methods are not able to remove the EEG noise completely and, in the worst case, may remove relevant information from the signal. Application of such methods prior to RESPERM or SEGMENTED might further improve the precision of change point detection and might be explored in further research. Hence, there are many attempts to extract single trial ERP information (e.g., [17]) from the EEG. The method suggested here can be applied to trace the time course of single trial ERPs over a relatively short time course. Here, we demonstrate this with data recorded in a face memory experiment where the N250 component of the ERP was tracked over repeated presentations of a target face that had to be recognized among other faces [8]. As shown by Tanaka et al. [7] and replicated by [8], on average, over a group of participants and when the time series is roughly split into two consecutive bins of a session, the N250 amplitude increased with target face familiarity. Therefore, it was of interest to apply RESPERM to determine a changepoint in the time series of single trials, demarcating the transition point between memory trace acquisition and asymptotic trace maintenance.

## 2. Materials and Methods

RESPERM as well as the dataset generation procedures were implemented in R language. The RESPERM algorithm uses the R *sample* function, which is based on a random number generation mechanism. The source code of RESPERM is publicly available on Github (https://github.com/gkonczak/chp.perm, accessed on 16 March 2022) and in Algorithm A1 in Appendix A.

For SEGMENTED we used the original procedure from the R package of Muggeo [9]. This procedure implements the bootstrap restarting algorithm to escape the local optima of the objective function when the segmented relationship is flat.

### 2.1. The Residuals Permutation-Based Method (RESPERM)

The proposed RESPERM method considers a discrete set (i.e., a grid) of possible changepoint locations. For each candidate changepoint, a set of parameters of a fitted changepoint regression model is determined. The finally selected changepoint optimizes the proposed criterion, that is, Cohen’s effect size [18], estimated using the permutation method. Moreover, our method allows for different variances in each regime. 

Formally, let us consider *n* observations denoted by $\left(x_{i},y_{i}\right)\;\mathrm{for}\;i=1,\;2,\;\ldots ,\;n$, divided into two subsets:

(4) $$S_{1}=\left\{\left(x_{i},y_{i}\right):i=1,\;2,\;\ldots ,\;k\right\}{\;\mathrm{and}\;S}_{2}=\left\{\left(x_{i},y_{i}\right):i=k+1,k+2,\;\ldots ,\;n\right\}$$

separated by a candidate changepoint $chp_{k}$ with *k* being from a set *K* = {s, s + 1, …, *n* *−* s} where *s* is the parameter for the RESPERM method. In our simulation study, *s* = 10, which follows from the minimal number of observations required to ensure the stability of linear regressions. 

We will consider two simple linear regression models with changepoint $chp_{k}$*:*

(5) $$y=\{\begin{array}{c} \beta_{01}+\beta_{11}x+\varepsilon_{1}\;,\;x\leq chp_{k}\\ \beta_{02}+\beta_{12}x+\varepsilon_{2}\;,\;x>chp_{k} \end{array}$$

where *y* is the dependent variable, *x* is the regressor, $\beta_{01}$, $\beta_{02}$, $\beta_{11}$ and $\beta_{12}$ are parameters of linear models and $\varepsilon_{1}$, $\varepsilon_{2}$ are error terms (noise). Based on the two subsets above, two regression lines with estimates of slopes ${\hat{\beta }}_{11}$ and ${\hat{\beta }}_{12}$ and intercepts ${\hat{\beta }}_{01}$ and ${\hat{\beta }}_{02}$ are fitted.

RESPERM aims to detect a change in the slope in linear regression models at the candidate changepoint $chp_{k}$, using Cohen’s effect size *d* [18]:

(6) $$d=(m_{A}-m_{B})\;/\;\sigma$$

where $m_{A}$ and $m_{B}$ are population means under consideration expressed in raw (original) measurement units and σ is the standard deviation of either population of measurements. 

In our case, in place of $m_{A},\;m_{B}$ and σ we use ${\hat{\beta }}_{11}$, ${\hat{\beta }}_{12}$ and the following estimate of the standard deviation σ:

(7) $$\sigma_{\beta }=\sqrt{\frac{\left(k-1\right)S_{\beta 11}^{2}+\left(n-k-1\right)S_{\beta 12}^{2}}{n-2}}$$

where $S_{\beta 11}\;\mathrm{and}\;S_{\beta 12}$ are the standard deviations of the $\beta_{11}$ and $\beta_{12}$ coefficients, respectively. Thus, to capture the effect size of slope change at the candidate changepoint $chp_{k}$, we use the adjusted Cohen’s *d* [18]:

(8) $$d_{k}=\left({\hat{\beta }}_{12}-{\hat{\beta }}_{11}\right)\;/\;\sigma_{\beta }$$

To estimate the unknown standard deviations $S_{\beta 11}\;\mathrm{and}\;S_{\beta 12}$ of the $\beta_{11}$ and $\beta_{12}$ coefficients, standard methods may not be reliable and a resampling method may offer improvement ([2,19,20]). We used the following permutation method (with *N*_perm_ = 1000 permutations): for each generated permutation of regression residuals, the coefficients ${\hat{\beta }}_{11}$, ${\hat{\beta }}_{12}$ and ${\hat{\beta }}_{01}$, ${\hat{\beta }}_{02}$ are estimated; then, based on the whole set of estimates obtained, the standard deviations $S_{\beta 11}\;\mathrm{and}\;S_{\beta 12}$ of the $\beta_{11}$ and $\beta_{12}$ coefficients are assessed.

We find the smallest observation number k^*^ which maximizes the adjusted Cohen’s $d_{k}$:

(9) $$k^{*}=\underset{k∊K}{\min}\left\{k:d\left(k\right)=\underset{k∊K}{\max}\;d\left(k\right)\right\}$$

The solution is the detected changepoint $chp=x_{k^{*}}$.

### 2.2. Monte Carlo Verification Setup

To examine the performance of RESPERM and to compare it with SEGMENTED, we performed a Monte Carlo simulation. A series of *n* = 100 observations with one changepoint were generated according to the following model:

(10) $$y=\{\begin{array}{c} 2+p\times \varepsilon_{j}\;,\;x\leq 50\\ 2+\left(x-50\right)+p\times q\times \varepsilon_{j}\;,\;x>50 \end{array}$$

where the covariate *x* = 1, 2, …, 100, $\varepsilon_{j}$ is the error term, coefficient *k* describes the level of noise (*p* = 3 major, *p =* 5 dominant) and coefficient *q* = 1 or *q =* 2/3 for the cases of equal or unequal variances in the two regimes. In all models considered, the variances in the two regimes (before and after the changepoint) were either equal (*q* = 1) or unequal (*q* = 2/3), that is, smaller after the changepoint than before. We considered four types of error distributions:

(1) $\varepsilon_{1}=\frac{1}{3}\varepsilon_{N}\;\left(\varepsilon_{N}\sim N\left(0,1\right)\right)$,
(2) $\varepsilon_{2}=\varepsilon_{U}-0.5\;\left(\varepsilon_{U}\sim U\left(0,1\right)\right)$,
(3) $\varepsilon_{3}=\varepsilon_{B22}-0.5\;\left(\varepsilon_{B22}\sim B\left(2,2\right)\right)$,
(4) $\varepsilon_{4}=\varepsilon_{B26}-0.25\;(\varepsilon_{B26}\sim B\left(2,6\right)$).

**Figure 1:**
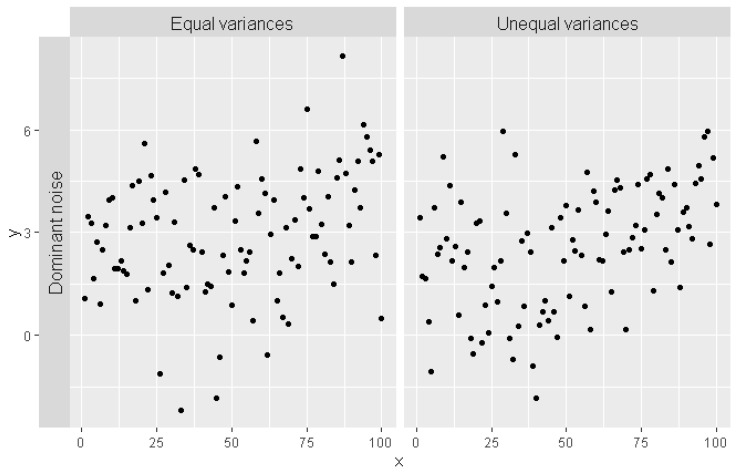
Typical generated time series with a single changepoint at *chp* = 50 for major noise level with normal error distributions, equal (**left**) and unequal (**right**) variances.

The error term $\varepsilon_{N}$ has standard normal distribution, $\;\varepsilon_{U}$ has uniform distribution in the [0, 1] interval, $\varepsilon_{B22}$ has beta distribution with shape parameters s_1_ = 2, s_2_ = 2 (symmetric) and $\varepsilon_{B26}$ has beta distribution with shape parameters s_1_ = 2, s_2_ = 6 (asymmetric). The expected values E(.) of the error distributions above are equal to zero and the variances $D^{2}\left(.\right)$ changes from 1/48 up to 1/9. The sample random series of observations for equal and unequal variance for major level of noise are shown in Figure 1.

In order to compare RESPERM with SEGMENTED, the following simulated datasets were created. The initial changepoint was established as *chp* = 50. For each combination of noise level, equal/unequal variances and four error distribution types mentioned above we generated *N* = 100 datasets according to formula (9) with proper values of coefficients *p* and *q* describing a given model and type of errors. First, the changepoint was estimated for each dataset using RESPERM. For each point *k* = 10, 11, …, 90 (assuming parameter *s* = 10), the variances of parameters of the linear models were estimated based on *N*_perm_ = 1000 permutations of residuals. The changepoint was estimated as a parameter k*, which maximizes Cohen’s *d* as in Formula (7).

We then estimated the changepoint for each dataset using SEGMENTED. The initial changepoint required in SEGMENTED (i.e., the parameter of the method) was chosen as the median of the regressor *x*.

To test the performance of both methods depending on the location of the changepoint *chp*, we also performed the procedures above described for different changepoints: *chp* = 4, 8, 12, 20, 30, 40, 50. In this *sensitivity analysis* we used only models with different variance (i.e., reduced in the 2-nd part of the time series), mainly because this case corresponds to the characteristics of EEG data. Moreover, for the changepoints implemented, only cases with normally distributed errors were considered. Two levels of noise were taken into account: major and dominant noise.

To quantitatively compare the performance of SEGMENTED and RESPERM for changepoint detection, we used the following measures. The first is the root-mean-square error (*RMSE*) given by

(11) $$RMSE=\sqrt{MSE}$$

where *MSE* is the mean square error estimated as:

(12) $$MSE\approx \frac{1}{N}\overset{N}{\underset{i=1}{\sum }}{\left(chp_{i}-chp\right)}^{2}$$

*chp* is a real (usually unknown), changepoint and $chp_{i}$ is a changepoint estimated by RESPERM or SEGMENTED.

The second measure is the relative bias (*RB*) of the estimated method as:

(13) $$RB\approx (\frac{1}{N}\sum_{i=1}^{N}chp_{i}-chp)/chp$$

We also used the standard deviation (*SD)*:

(14) $$SD=\sqrt{VAR}$$

where the variance *VAR* is estimated as:

(15) $$VAR\approx \frac{1}{N}\sum_{i=1}^{N}{\left(chp_{i}-\bar{chp}\right)}^{2},\;\bar{chp}=\frac{1}{N}\sum_{i=1}^{N}chp_{i}$$

### 2.3. Single-Trial ERP Data

Details of the experimental setup, data acquisition and preprocessing procedures for the single-trial ERP data and conventional ERPs from 16 participants have been reported by [8]. 

In short, the task was to identify a designated face among a set of 12 different faces by means of a button press. The target face was presented in 72 trials, randomly interspersed in 792 nontarget trials with a mean interval of 12 trials between the target face presentations. Each trial took 2 s. Trials without correct answers were discarded from analysis but considered in the timeline.

EEG was recorded from 64 electrodes and segmented into epochs of 1 s with a 100-ms prestimulus baseline. The EEG signals were digitally low-pass filtered at 40 Hz, down-sampled to 250 Hz, re-calculated to common average reference and cleaned from ocular artifacts. Here, we consider only single trial amplitudes of the N250 component in response to target faces, extracted from the spatially averaged EEG within a region of interest across 6 right hemisphere electrodes (TP10, P8, P10, PO8, PO10, O2) from 230 to 320 ms after face onset. This region and interval of interest were based on the original study [7]. Epochs with activity ranges > 100 µV within any channel were discarded from analysis.

## 3. Results

### 3.1. Monte Carlo Simulations

Table 1 and Table 2 present the *RMSE* and *RB* (with corresponding *SD*), respectively, of changepoint estimations with *chp =* 50, obtained by RESPERM and SEGMENTED for two levels of noise, four error distribution types and equal and unequal variances. *RMSE* of changepoints in time series with minor noise was smaller by up to 50% for RESPERM than SEGMENTED in all cases with equal variances and in 3 of four cases with unequal noise; only for the beta asymmetric distribution of noise *RMSE* was smaller for SEGMENTED. For time series with major noise and all error types, *RMSE*s of changepoint location estimates were smaller (unequal variances) or even noticeably smaller (equal variances) for RESPERM. In all cases of dominant noise, *RMSE*s of changepoint estimation were noticeably smaller for RESPERM. Thus, RESPERM is consistently more precise than SEGMENTED for major and dominant levels of noise.

As shown in Table 2, relative bias was very small and, compared to the large *SD*s, especially for SEGMENTED, negligible. Taking into account the much larger *RMSE*s for SEGMENTED as compared to RESPERM, especially for major and dominant noise, we conclude that RESPERM shows greater accuracy (precision) than SEGMENTED.

**Figure 2:**
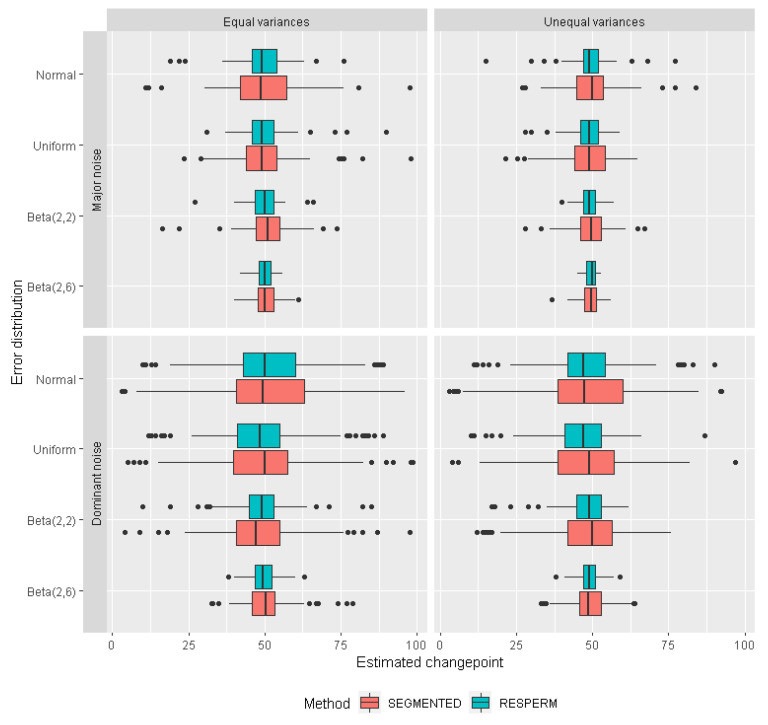
Distributions of changepoint estimates with RESPERM and SEGMENTED methods in simulated data with *chp* = 50 for two levels of noise with equal and unequal variances.

The box-whisker plots in Figure 2 visualize the distributions of the changepoint estimates obtained from the simulations, showing the medians and inter-quartile ranges (IQR; box width). Whiskers represent data falling outside the IQR; outliers are depicted as points outside of the whiskers.

In most cases, the changepoint estimate distributions are slightly asymmetric. There are almost no differences between the medians (centers) obtained with the two methods. Only in a few cases there are small differences: for major noise, beta-symmetrically distributed errors for both equal and unequal variances, for normally distributed errors for unequal variances, for dominant noise, uniformly distributed errors and for unequal variances and beta-symmetrically distributed errors with equal variances. Since there are no significant differences in the location of medians, on average, the two methods provide very similar median changepoint. 

In contrast to the medians, the IQRs in cases of major and dominant noise are noticeable smaller for RESPERM than for SEGMENTED for all error distribution types and both equal and unequal variances. The variation in changepoint estimates outside the IQR is also much smaller for RESPERM than for SEGMENTED at all levels of noise and for both equal and unequal variances; the only exception is for asymmetric beta-distributed errors and unequal variances. For major and dominant levels of noise, this difference is substantial. Hence, the results from SEGMENTED show a wider spread than those for RESPERM, indicating higher accuracy of RESPERM.

To check the similarity of the estimation results of both methods, Pearson correlation coefficients for changepoint estimates from SEGMENTED and RESPERM were calculated. Table 3 presents Pearson correlation coefficients for simulations with *chp* = 50 for the two levels of noise, four error distribution types and equal and unequal variances. 

As shown in Table 3, the estimated changepoints obtained with the two methods are always positively correlated, ranging from moderate (*r* = 0.42) to strong (*r* = 0.87). This supports the general similarity of the results but with non-negligible differences in some cases.

**Figure 3:**
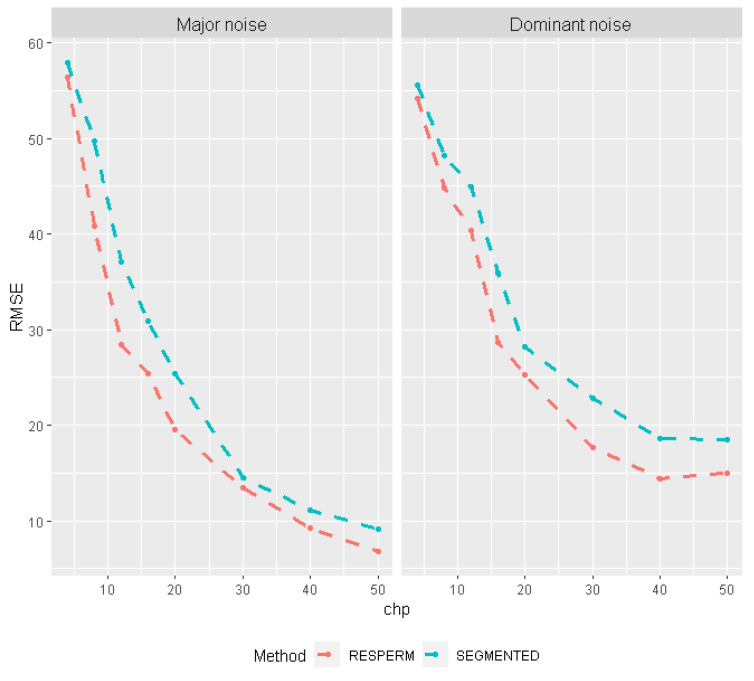
*RMSE* of change point estimates as a function of simulated changepoint location for RESPERM and SEGMENTED for major and dominant noise with normal distributions.

The results of the sensitivity analysis (i.e., the dependence of *RMSE* on the location of changepoint) are shown in Figure 3. In the case of greater variance of random errors (minor and dominant), RESPERM yields consistently smaller RMSE than SEGMENTED for all simulated changepoints. Overall, higher levels of random errors lead to a larger *RMSE* in the changepoint assessment but less so for RESPERM than SEGMENTED.

Moreover, it should be noted that in many cases (especially for *chp* = 4, 8 and 12) SEGMENTED did not yield change points, forcing us to rerun the analysis multiple times.

### 3.2. Application to Single Trial ERP Data from a Face Memory Task

**Figure 4:**
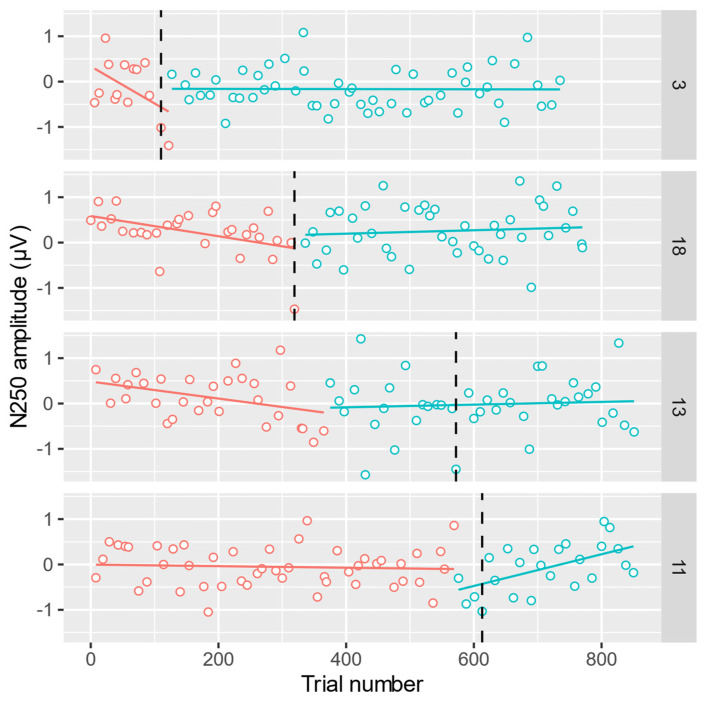
Time series of N250 amplitudes for selected participants 3, 18, 13, 11 with regression lines drawn before and after the changepoint detected by RESPERM (*chp^res^*). The SEGMENTED-detected changepoints (*chp^seg^*) are marked by vertical dashed lines.

**Figure 5:**
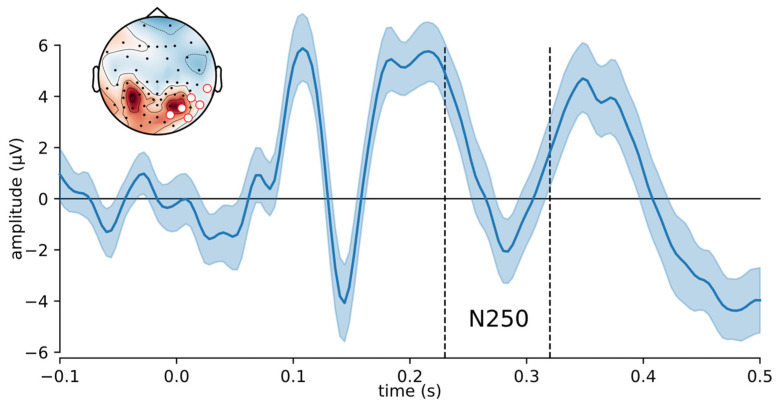
ERP waveform (±95% CI) averaged over all Joe trials and over all electrodes of interest for Participant 11 with dashed vertical lines indicating the N250 component time window. The electrodes of interest (red circles) and the distribution of scalp potentials in N250 time window are presented in the top left corner.

Figure 4 shows the timeseries of the N250 component amplitudes elicited by correctly recognized target faces for 4 selected participants from [8]. Each point in Figure 4 is an amplitude measure for a single experimental target trial of a single participant measured in the average over 6 electrodes of interest (TP10, P8, P10, PO8, PO10, O2) and during a 230–320 ms post-stimulus time window. These N250-related electrodes and time window are shown in Figure 5 together with an ERP waveform averaged over all target trials for Participant 11. Grand-averaged ERP waveforms are available in [8]. We assume that face learning or familiarization proceeds in two phases, that is, initial acquisition, represented by a steep increase of N250 across trials, followed by consolidation or retention where N250 is relatively large and more stable. Hence, we aimed to find a changepoint between two linear regression lines during participants’ face learning using RESPERM and SEGMENTED, respectively. The latency of this changepoint may be an indicator of the transition point, demarcating the speed of the development of a mental representation of the target face.

The inherent characteristic of both RESPERM and SEGMENTED is the random factor described in the Methods section above. Hence, in order to apply the methods to the analysis of noisy EEG data, we ran each method 100 times and selected the optimal solution in terms of the method-specific optimization function—maximum Cohen’s *d* for RESPERM or minimum residual sum of squares (*RSS*) for SEGMENTED. The detailed results are given in Table 4. The observation numbers from RESPERM corresponds to trial numbers in our EEG experiment. In RESPERM, the detected changepoint *k^res^* is the observation number and *chp^res^* is the trial number corresponding to *k^res^*. The detected changepoint *k^seg^* is the closest observation number corresponding to the solution *chp^seg^* (rounded to the nearest integer) found by SEGMENTED. SEGMENTED was unable to find a solution for Participant 4.

The mean absolute difference of changepoints between both methods was 64 trials (*SD* = 71), counting also the nontarget trials, with the largest difference for Participant 13 (207 trials) and no difference for Participant 18. There is a strong Pearson correlation (*r* = 0.86) between the changepoints found by both methods: *chp^res^* and *chp^seg^.* Hence, results from RESPERM and SEGMENTED are in a good agreement, conforming with the simulations presented in Section 3.1.

## 4. Discussion and Conclusions

We considered the problem of changepoint detection in linear regression models based on noisy data and devised a new method (RESPERM), based on permutation-based residuals and systematically compared its results with those of the often-recommended SEGMENTED method. RESPERM maximizes Cohen’s effect size *d* with the parameters estimated by the permutation of residuals in the linear model. RESPERM was compared with SEGMENTED in a number of simulations and in an application to single trial ERP component amplitudes from a face learning experiment. In the simulation study, four variants of noise were considered from normal and beta distributions together with equal and unequal variances and different distributions of random errors were taken into account.

In case of minor noise, that is, for relatively clean data, in most cases SEGMENTED yielded smaller standard errors than RESPERM. However, for noisier data, that is, in cases of major and dominant greater variance of random errors, RESPERM was consistently superior and characterized by a smaller standard error of the changepoint estimates than SEGMENTED for all changepoints, that is, independent of their position in a time series. Therefore, RESPERM appears more precise than SEGMENTED for noisier data.

Noisy data are typical for many applied situations, for example in ERP analyses with single trials or small averages. It is difficult with any method to find changepoints in highly noisy data, especially if they are located at the initial or terminal positions of the time series. The greater precision of RESPERM at such early points justifies recommending it for such difficult situations.

Our application of RESPERM to single-trial ERPs was based on previous group evidence that the averaged N250 amplitude increases from the initial portion of repeated faces presentations to the later portion [7,8]. Hence, we assumed that face learning in individual participants may be conceived to often consist of two, approximately linear trends with an initial dynamic acquisition phase reflected in an increase of N250 followed by consolidation or retention, where the N250 amplitude is large and stable. These assumptions are likely only approximations to more curvy-linear trajectories. Nevertheless, this approximation constitutes a progress over the standard approach of averaging ERPs over blocks of learning. The present approach allowed us to identify change points that differ between individuals. In perspective, the application to single trial ERP, based on relatively simple amplitude measures in a region of interest, could be combined with more sophisticated approaches such as multivariate pattern analysis (e.g., [21]).

The similar outcomes of the RESPERM and SEGMENTED methods demonstrate the stability of the basic approach and shows that changepoint detection is suitable to investigate the dynamics of face learning at a single-participant level. On a more general level, changepoint detection is used to investigate many phenomena where a dynamic change over time is of interest. Especially when such dynamics are fast or when data are noisy, RESPERM appears to be a good choice and applicable to data from many fields of research, including medical research.

## Figures and Tables

**Table 1:** *RMSE* for change point estimations with *chp* = 50 for two levels of noise, four error distribution types and equal and unequal variances.

Error Distribution	Noise Level	Equal Variances		Unequal Variances	
		SEGMENTED	RESPERM	SEGMENTED	RESPERM
Normal	Major	12.96	7.88	9.16	6.88
	Dominant	20.48	17.38	18.51	14.94
Uniform	Major	11.09	7.71	9.04	5.89
	Dominant	19.55	15.35	16.42	14.16
Beta (2,2)	Major	8.12	4.63	6.12	3.30
	Dominant	15.43	10.39	12.51	8.79
Beta (2,6)	Major	4.10	2.75	3.52	2.06
	Dominant	8.10	4.17	6.59	3.62

**Table 2:** Relative bias (*RB*, in %) and *SD* for change point estimations with *chp* = 50 for two levels of noise, four error distribution types and equal/unequal variances.

Errors Distribution	Noise Level	Equal Variances		Unequal Variances	
		SEGMENTED	RESPERM	SEGMENTED	RESPERM
		*RB/SD*	*RB/SD*	*RB/SD*	*RB/SD*
Normal	Major	0.12/12.96	−1.26/7.86	−1.60/9.13	−2.08/6.80
	Dominant	1.02/20.47	0.64/17.38	−3.84/18.41	−5.78/14.66
Uniform	Major	−0.66/11.08	−0.20/7.71	−3.16/8.90	−3.84/5.57
	Dominant	−1.08/19.54	−1.72/15.32	−5.28/16.21	−9.68/13.30
Beta (2,2)	Major	1.66/8.07	0.32/4.63	−1.32/6.09	−2.20/3.11
	Dominant	−3.02/15.36	−1.88/10.35	−3.30/12.40	−4.42/8.51
Beta (2,6)	Major	0.68/4.09	0.42/2.74	−1.58/3.43	−1.44/1.93
	Dominant	1.80/8.05	−0.54/4.16	−2.56/6.46	−1.80/3.51

**Table 3:** Pearson correlation coefficients for changepoint estimates from SEGMENTED and RESPERM (with *chp* = 50).

Error Distribution Type	Major Noise		Dominant Noise	
	eV	ueV	eV	ueV
Normal	0.59	0.75	0.83	0.46
Uniform	0.82	0.61	0.66	0.42
Beta (2,2)	0.81	0.79	0.80	0.69
Beta (2,6)	0.84	0.67	0.77	0.87

Note: eV—equal variances, ueV—unequal variances.

**Table 4:** RESPERM- and SEGMENTED-detected changepoints of the N250 amplitudes across trials for 16 participants, sorted by RESPERM latencies (*chp^res^*).

	RESPERM			SEGMENTED	
Participant Number	*d*	*k^res^*	*chp^res^*	*k^seg^*	*chp^seg^*
3	3.556	14	122	13	110
6	6.250	12	139	10	114
2	4.636	16	179	15	165
15	3.791	17	188	12	136
17	4.512	17	208	14	172
9	5.340	21	235	20	226
20	2.088	24	282	29	334
14	1.358	10	284	27	486
18	3.631	29	319	29	319
5	4.520	28	335	26	305
13	3.120	30	365	48	572
19	4.563	45	370	22	177
7	5.781	35	389	34	378
11	5.089	48	569	52	613
12	4.029	50	578	57	657
4	2.058	57	673	-	-

*d*: the adjusted Cohen’s effect size *d* for RESPERM. *k^res^* and *k^seg^*: observation numbers corresponding to changepoints (see explanation in text). *chp^res^* and *chp^seg^*: trial number for RESPERM and direct solution by SEGMENTED rounded to the nearest integer.

## Data Availability

The data used in single-trial ERP experiments are publicly available at https://osf.io/7x6w5/ (accessed on 16 March 2022).

## Appendix A

**Algorithm A1:** The code of RESPERM implemented in R.
res.perm <- function(x,y,N_perm=100) { n = length(x) first_k = 10 last_k = n − 10 Cohen_d = rep(NA,n) simple.fit = lm(y~x) res = simple.fit$residuals yf = simple.fit$fitted if (N_perm < 100) stop(“Too few permutations”) if (n < 50) stop(“Too few observations”) ### Finding the greatest value of the vector Cohen_d for (k in first_k:last_k) { simple1.fit = lm(y[1:k]~x[1:k]) simple2.fit = lm(y[(k+1):n]~x[(k+1):n]) b1 = simple1.fit$coefficients[2] b2 = simple2.fit$coefficients[2] b1s = c(); b2s = c() for (i in 1:N_perm) { ys = yf + sample(res) b1s[i] = lm(ys[1:k]~x[1:k])$coefficients[2] b2s[i] = lm(ys[(k + 1):n]~x[(k + 1):n])$coefficients[2] } Cohen_d[k] = (b2 − b1)/sqrt(((k − 1)*var(b1s)+(n − k − 1)*var(b2s))/(n − 2)) } d = max(Cohen_d[first_k:last_k], na.rm =T) k_star = order(Cohen_d[first_k:last_k],decreasing = T)[1] + first_k−1 return(list(“k_star” = k_star, “chp” = x[k_star], “d” = d)) }
